# Sub-inhibitory concentrations of oxacillin modify the expression of *agr* locus in *Staphylococcus aureus* clinical strains belonging to different clonal complexes

**DOI:** 10.1186/s12879-018-3088-7

**Published:** 2018-04-16

**Authors:** Esther Viedma, Dafne Pérez-Montarelo, Jennifer Villa, Irene Muñoz-Gallego, Nieves Larrosa, Nuria Fernández-Hidalgo, Joan Gavaldà, Benito Almirante, Fernando Chaves

**Affiliations:** 1 Department of Microbiology, Hospital Universitario 12 de Octubre, Madrid, Spain; 2 Department of Infectious Diseases, Hospital Universitari Vall d’Hebron, Barcelona, Spain

**Keywords:** *Staphylococcus aureus*, Oxacillin, Sub-inhibitory concentrations, *agr* locus expression, *agr* mutations, Clonal complex

## Abstract

**Background:**

The ability of *Staphylococcus aureus* to invade tissues and cause an infectious disease is the result of a multi-factorial process supported by the huge number of virulence factors inherent to this microorganism tightly regulated by the accessory gene regulator (*agr*). During antimicrobial therapy bacteria may be exposed to sub-inhibitory concentrations (subMICs) of antibiotics that may trigger transcriptional changes that may have an impact on the pathogenesis of infection. The objective of this study was to investigate the effect of oxacillin sub-MICs on *agr* system expression as the key component in the regulation of virulence in methicillin-susceptible (MSSA) and -resistant *S. aureus* (MRSA) strains. Furthermore, we studied the genetic basis of the *agr* locus and their potential association with the expression levels.

**Methods:**

We have examined the expression of *RNAIII* and *agrA* mRNA as biomarkers for *agr* expression in the presence and absence of oxacillin subMICs in 10 MSSA and 4 MRSA clinical strains belonging to 5 clonal complexes (CC45-*agrI*, CC8-*agrI*, CC5-*agrII*, CC15-*agrII* and CC30-*agrIII*) causing endovascular complications. The DNA sequences of *agr* locus were obtained by whole genome sequencing.

**Results:**

Our results revealed that exposure to subMICs of oxacillin had an impact on *agr* locus expression modifying the relative levels of expression with increases in 11 strains and with decreases in 3 strains. Thereby, the exposure to subMICs of oxacillin resulted in higher levels of expression of *agr* in CC15 and CC45 and lower levels in CC30. We also observed the presence of mutations in *agrC* and *agrA* in 13/14 strains with similar mutation profiles among strains within individual CCs except for strains of CC5. Although, *agr* expression levels differed among strains within CCs, the presence of these mutations was associated with differences in *agr* expression levels in most cases.

**Conclusions:**

Changes in *agr* expression induced by exposure to oxacillin subMICs should be considered because they could lead to changes in the virulence modulation and have an adverse effect on the course of infection, especially in certain clonal complexes.

**Electronic supplementary material:**

The online version of this article (10.1186/s12879-018-3088-7) contains supplementary material, which is available to authorized users.

## Background

*Staphylococcus aureus* remains the major cause of endovascular infections with high morbidity and mortality rates that may reach 40% in case of infective endocarditis [1]. Even though most *S. aureus* genotypes exhibit the capacity to cause invasive disease, several studies have reported the association of particular clonal complexes (CC5, CC15 and CC30) with endovascular complications such as infective endocarditis [2–4].

The ability of *S. aureus* to produce infection involves a wide variety of virulence determinants, such as exotoxins, adhesins and immune evasion genes, constituting a gene framework that is under the control of global regulators, most notably the quorum-sensing accessory gene regulator (*agr*). Under conditions of high cell density, a*gr* is responsible for the increased expression of many toxins and degradative exoenzymes, and decreased expression of several colonization factors. This regulation is important for the timing of virulence factor expression during infection [5]. Thereby, when adhesion to host tissue is crucial, cell density and *agr* expression are low, resulting in an increase in the surface virulence factors that are required for the colonization process. Once infection is established, the increased cell density results in higher *agr* expression that leads to production of toxins which trigger the host inflammatory response [6, 7]. The *agr* system up-regulates the production of secreted virulence factors such as exotoxins, and down-regulates the production of cell associated virulence factors [5, 8–10].

The *agr* locus consists of two divergent transcriptional loci, *RNAII* and *RNAIII*, driven by promoters P2 and P3, respectively [8, 9]. The P2 operon encodes four genes, *agrB, agrD, agrC* and *agrA*. The *agrA* and *agrC* genes constitute a two-component signaling system, of which *agrC* is the receptor, and *agrA* is the response regulator [11, 12]. Upon activation by *agrC*-dependent phosphorylation, agrA binds to the P2 promoter region for *RNAII* and the P3 promoter region for *RNAIII*, the effector of target gene regulation that also encodes delta-haemolysin, the expression of which serves as a surrogate for *agr* functionality [13, 14]. Moreover *agrA* directly up-regulates transcription of the phenol-soluble modulins operon (*psmα* and *psmβ*) whose role in the pro-inflammatory response has already been demonstrated [6, 7, 12, 15]. Several studies have reported that different mutations in the *agr* operon, mainly within the *agrC* or *agrA* genes, lead to decreased *RNAIII* expression and reduced virulence [9, 16–18]. However, it is known that strains with dysfunctional *agr* are more likely to cause persistent infection and be associated with poor outcome [19–21].

Despite the emergence of methicillin resistant *S. aureus* (MRSA), beta-lactams and specifically antistaphylococcal penicillins (nafcillin, oxacillin, cloxacillin and dicloxacillin), remain the usual treatment for the management of invasive infections caused by methicillin-susceptible *S. aureus* (MSSA). However, because of impaired diffusion and distribution of antibiotic, not all bacteria are exposed to the required lethal concentrations of bactericidal agents and therefore are likely subjected to sub-MIC antibiotic effects on the expression of virulence factors [7, 22–30]. Thereby, the effect of subMICs oxacillin on phenol-soluble modulins and *RNAIII* have been studied [27, 28], showing a decrease in mRNA level in both virulence determinants in USA300 strain. However, increase in *spa* and *lukE* mRNA levels under exposure to subMICs of oxacillin, cephalotin and penicillin were reported by Subrt et al. [29]. Moreover, Rasigade et al. revealed that subMIC of oxacillin, moxifloxacin and linezolid led to increase *fnbA/B* mRNA levels [30]. Collectively all this studies add more evidence that, that suboptimal concentrations of different antibiotics modulate the expression of virulence factors in *S. aureus,* and therefore may exert an influence over the pathogenesis of infection [23]. Nevertheless, limited data exist showing the effect of oxacillin in MSSA clinical strains. Thereby, the aim of the present study was to investigate the impact of sub-inhibitory concentrations (subMICs) of oxacillin on the *agr* operon expression, as a key element of virulence regulatory network, in a collection of *S. aureus* clinical strains belonging to the main clonal complexes related with invasive diseases. In addition, we analyzed whether the presence of mutations in *agr* locus could be associated with differences in *agr* gene expression.

## Methods

### *S. aureus* strains collection

A total of 14 *S. aureus* strains (10 MSSA and 4 MRSA) from two multi-center investigations of infective endocarditis [31] and catheter-related bacteremia [32] were included in the present study. The strains were selected as representatives of the CCs that were most frequently detected in the original investigations: CC5 (ST5 and ST125), CC15, CC30, CC45 and CC8 (ST8 and ST72). We randomly selected two MSSA strains of each CC, as well as methicillin resistant (MRSA) strains that belonged to the same CCs if they were available (Table 1).

**Table 1:** Clinical and genotypic characteristics of *S. aureus* strains and suboptimal concentration of oxacillin used in this study

Strain	Clinical diagnosis	Clonal Complex	*Agr* group	*MecA*	MIC oxacillin (mg/L)	sub_MIC oxacillin (mg/L)
SA_123	Infective endocarditis	CC45	I	Negative	0.38	0.02
SA_520	Infective endocarditis	CC45	I	Negative	0.50	0.03
SA_170015	Infective endocarditis	CC45	I	Positive	1.00	0.13
SA_180015	Infective endocarditis	CC8_ST8	I	Negative	0.25	0.03
SA_190006	Infective endocarditis	CC8_ST72	I	Negative	1.00	0.03
SA_70002	Infective endocarditis	CC8_ST72	I	Positive	2.00	0.13
SA_80004	Infective endocarditis	CC5_ST5	II	Negative	0.50	0.05
SA_103	Infective endocarditis	CC5_ST5	II	Negative	0.50	0.03
SA_170006	Infective endocarditis	CC5_ST125	II	Positive	256	8.00
SA_180009	Infective endocarditis	CC5_ST125	II	Positive	2.00	0.50
SA_10009	Infective endocarditis	CC15	II	Negative	0.38	0.19
SA_10014	Infective endocarditis	CC15	II	Negative	0.50	0.13
SA_80001	Infective endocarditis	CC30	III	Negative	0.25	0.05
SA_107	Catheter related bacteremia with septic complications	CC30	III	Negative	0.25	0.06

*MIC* minimal inhibition concentration

### Sub-inhibitory concentrations of oxacillin and growth curves

Strains were exposed to subMICs of oxacillin in order to observe *agr* gene expression under this condition. To select the optimal subMIC for each strain, we monitored growth by measuring the optical density of broth cultures exposed to ½, ¼ and 1/8 of the respective MIC for each strain as previously determined by E-test (bioMérieux). We defined the subMIC as the highest concentration below the MIC that showed no major growth defects over the entire growth curve relative to a control culture without oxacillin. [27] For each strain, the MIC and subMIC of oxacillin are shown in Table 1. Oxacillin was added to cultures at the time of inoculation (1:100 from pre-cultures) into 10 ml of tryptic soy broth (TSB) and incubated at 37 °C in a shaking incubator at 250 rpm. Growth under the same conditions was monitored by determining the optical density at 600 nm (OD600) and it was measured at the end of the incubation to determine the impact of antibiotic exposure over bacterial density (Additional file 1: Figure S1).

### RNA isolation

Isolated colonies were resuspended in TSB and adjusted to 0.5 McFarland and incubated at 37 °C overnight. Fresh TSB (10 ml in 50-ml conical flask) was inoculated with 100 μl of overnight broth cultures (1:100 dilution) and incubated at 37 °C in a shaking incubator at 250 rpm in the presence or absence of the appropriate subMIC of oxacillin. Growth was monitored by determining the optical density (OD600). RNA was isolated after growth for 20 h (stationary phase) using the Qiagen Rneasy Mini kit according to the manufacturer’s instructions and after addition of 2 volumes of RNA protect reagent (Qiagen, N.V).

### Reverse transcription and qRT-PCR

Expression of both the *agrA* and *RNAIII* genes was analyzed by quantitative reverse transcriptase PCR (qRT–PCR) as surrogate biomarkers of global *agr* operon activation. Expression levels of the housekeeping *gyrB* gene were used to normalize differences in mRNA quantification. In brief, 1 μg of total RNA was transcribed into cDNA using a Transcriptor First Strand cDNA Synthesis Kit (Roche Applied Science). Quantitative real time PCR was carried out on a LightCycler® 480 with LightCycler® 480 FastStart DNA Master Plus SYBR Green I (both Roche Applied Science). Previously described primers were used to amplify the *RNAIII*, *agrA* and *gyrB* transcripts [28, 33]. The experiments were performed using three biological replicates, each tested in triplicate. For each reaction, the ratio of the target gene (*RNAIII* or *agrA*) to *gyrB* transcripts was calculated based on the difference in cycle thresholds (2^Ct_*gyrB*-Ct_target_gene^) for all strains to obtain *RNAIII* and *agrA* relative gene expression levels in stationary phase in the presence and absence of the subMIC of oxacillin [33, 34]. The results were expressed as the *n-fold* difference in levels of transcription of the 2^Ct_*gyrB*oxa-Ct_*agrA*oxa^ and 2^Ct_*gyrB*oxa-Ct_*RNAIII*oxa^ relative to 2^Ct_*gyrB*control_Ct_*agrA*control^ and 2^Ct_gyrB*control*-Ct_*RNAIII*control^, respectively.

### Genotyping analysis of *agr* locus by whole genome sequencing

The DNA sequences of *agr* locus were obtained after that assembling of reads obtained from whole genome sequencing of all strains included in this study before of exposure to oxacillin subMICs. The sequencing was performed on the Illumina MiSeq platform (150 bp paired-end reads) using TruSeq DNA PCR free kits (Illumina, CA, USA). These *agr* nucleotide sequences of the entire *agr* locus were compared with those of *agr* group reference strains -*agr I:* NCTC 8325, *agr II*: N315; *agr III*: MW2- by multiple alignments using the Geneious server (Geneious 10.0.6).

### Statistical analysis

In order to yield more accurate and reliable summary statistics continuous variables were expressed as mean and standard deviation (SD) from the expression data obtained of three biological replicates each tested in triplicate. These values were analyzed by Student’s t or Wilcoxon test, as appropriate. Significance was defined as *p* < 0.05. Data were stored and analyzed using SPSS software version 15.0 (SPSS, Chicago, IL, USA).

### Nucleotide sequence accession number

The nucleotide sequences were deposited in GenBank (NCBI) under the accession numbers: MKYX00000000, MKYY00000000, MKYZ00000000, MKZA00000000, MKZB00000000, MKZC00000000, MKZD00000000 MKZE00000000, MKZF00000000, MKZG00000000, MKZH00000000, MKZI00000000, MKZJ00000000 and MKZK00000000.

## Results

### Expression of *agr* locus in the presence of oxacillin subMICs in stationary phase and its relationship with the CC, *agr* type and methicillin resistance

**Fig. 1:**
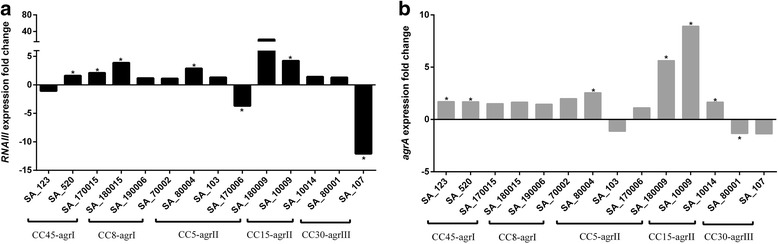
Effect of subMICs of oxacillin over *RNAIII* (**a**) and *agrA* (**b**) expression levels under oxacillin subMICs exposure in stationary growth phase. The expression levels of both *agrA* and *RNAIII* genes were determined by qRT–PCR experiments (three different experiments). The results are expressed as the n-fold variation of *RNAIII* (A) and *agrA* (B). An asterisk indicates a significant difference (*P* < 0.05)

The exposure to subMICs of oxacillin in stationary phase resulted in a trend to higher *RNAIII* transcript levels than in absence of the antimicrobial agent for 11/14 strains being statistically significant for 5 strains of them (*p* < 0.05) (Fig. 1). Only SA_123 (CC45) (p: 0.735), SA_107 (CC30) (p: 0.028) and SA_170006 (CC5) (p: 0.043) exhibited reduced *RNAIII* expression (Table 2). We observed a similar pattern of relative gene expression for *agrA*, with increased levels of *agrA* mRNA in the presence of oxacillin subMICs for all strains, being statistically significant for 5 of them (p < 0.05), except SA_80001 (CC30) (p: 0.028), SA_107 (CC30) (p: 0.345) and SA_103 (CC5) (p: 0.465) which showed lower levels of expression (Fig. 1, Table 2, Additional file 2: Table S1). In addition, we observed that in four strains (SA_123, SA_103, SA_170006 and SA_80001), the change in the expression of *RNAIII* and *agrA* did not move in the same direction. However, these differences in the expression values for both *agrA* and *RNAIII*, in the presence and absence of oxacillin subMICs, were not statistically significant.

**Table 2:** Expression levels changes in *RNAIII* and *agrA* locus under the exposure to oxacillin subMICs in the stationary growth phase

	*RNAIII*			*agrA*		
Strain	subMIC OXA^a^ vs. No subMIC OXA Fold_Change		P ^b;c^	subMIC OXA vs. No subMIC OXA Fold_Change		P
SA_123	1.08	NEG	0.735	1.71	POS	0.128
SA_520	1.62	POS	**0.028**	1.69	POS	**0.043**
SA_170015	2.11	POS	**0.046**	1.51	POS	0.116
SA_180015	3.88	POS	**0.028**	1.65	POS	0.138
SA_190006	1.19	POS	0.465	1.46	POS	0.080
SA_70002	1.12	POS	0.144	1.99	POS	0.144
SA_80004	2.90	POS	**0.018**	2.55	POS	**0.046**
SA_103	1.32	POS	0.465	1.13	NEG	0.465
SA_170006	3.69	NEG	**0.043**	1.12	POS	0.398
SA_180009	20.58	POS	0.068	5.63	POS	**0.028**
SA_10009	4.23	POS	**0.012**	8.91	POS	**0.028**
SA_10014	1.43	POS	0.091	1.65	POS	**0.018**
SA_80001	1.31	POS	0.249	1.34	NEG	**0.028**
SA_107	12.08	NEG	**0.028**	1.37	NEG	0.345

^a^ OXA: Oxacillin

^b^ POS: fold change positive in the expression level under exposure to oxacillin subMIC. NEG: fold change negative in the expression level under exposure to oxacillin subMICs

^c^ Bold numbers indicate statistical significant results (*P* < 0.05)

In order to investigate the association between *agr* locus expression and methicillin resistance profile we studied the *agr* locus expression in the strains classified as MSSA and MRSA and we found higher *agrA* and *RNAIII* levels of gene expression for both groups in the presence of subMICs of oxacillin than in absence of this antibiotic, although this trend was not statistically significant (Table 3). When we analyzed by CC, we detected an increase in relative gene expression for *RNAIII* and *agrA* in the presence of oxacillin for both CC45 (*p* = 0.005 and *p* = 0.000, respectively) and CC15 (p = 0.005 and *p* = 0.001, respectively). Nevertheless, CC30 showed a decrease in gene expression for both *RNAIII* and *agrA* in the presence of subMICs of oxacillin, but the difference was not statistically significant.

**Table 3:** Expression levels changes in *RNAIII* and *agrA* locus under exposure to oxacillin subMICs in the stationary growth phase clustered by methicillin resistance; Clonal Complex and *agr* type

	*RNAIII*			*agrA*		
	subMIC OXA ^a^ vs. No subMIC OXA Fold_Change		P ^b;c^	subMIC OXA vs. No subMIC OXA Fold_Change		P
Methicillin resistance						
MRSA	2.26	POS	0.051	1.80	POS	0.163
MSSA	1.75	POS	0.149	1.82	POS	**0.003**
Clonal complex						
CC45	1.74	POS	**0.005**	1.61	POS	**0.000**
CC8	1.07	NEG	1.000	1.81	POS	**0.022**
CC5	2.36	POS	0.108	3.61	POS	**0.008**
CC15	3.48	POS	**0.005**	4.48	POS	**0.001**
CC30	7.16	NEG	0.071	1.32	NEG	0.138
*agr* type						
*agrI*	1.10	POS	0.804	1.62	POS	**0.001**
*agrII*	3.95	POS	**0.001**	3.98	POS	**0.000**
*agrIII*	7.16	NEG	0.071	1.32	NEG	0.138

^a^ OXA: Oxacillin

^b^ POS: fold change positive in the expression level under exposure to oxacillin subMIC. NEG: fold change negative in the expression level under exposure to oxacillin subMICs

^c^ Bold numbers indicate statistical significant results (*P* < 0.05)

With regard to *agr* type, *agrII* strains showed higher expression levels under oxacillin exposure for both *RNAIII* and *agrA* (Table 3).

### Genotypic analysis of *agr* locus

After observing differences in the *agr* expression among the strains, we intended to know whether these strains harbored any mutations that might potentially be responsible for the *agr* functionality. In general, sequencing of the *agr* locus revealed similar mutation profiles among strains belonging to the same CC (Table 3). Strains from CC45-*agrI* showed up to 9 non-synonymous mutations in the *agrB* region and from 2 to 3 non-synonymous mutations in *agrC*, including missense mutations in SA_123 (417 Glu > Lys) and SA_170015 (103, Ile > Thr). Strains belonging to CC8-*agrI* exhibited mutations exclusively in the *agrC* and *agrA* genes. In the coding region of *agrC*, SA_190006 and SA_70002, displayed the same three non-synonymous mutations (42 Val > Gly; 262 Asp>Ala; 258 Phe > Ile). None of these mutations were present in SA_180015, although a frameshift deletion was detected (a409-1 bp, 138Lys/del). All CC5-*agrII* strains except SA_180009 displayed mutations: a single non-synonymous mutation (96, Ser > Leu) in SA_103, a frameshift one base pair deletion at position 313 in SA_80004, and a non-synonymous mutation at position 121 (121, Ile > Thr) in SA_170006. The two strains from CC15-*agrII* did not show any differences between them, with both exhibiting the same missense substitution at position 136 in *agrA* (136, Lys > Arg). No differences were found between the two CC30-*agrIII* strains, which showed the same genotypic profile with missense mutations in the coding region of *agrB* (49 Ile > Leu and 115 Ile > Leu) and *agrC* (55 Gly > Arg) (Table 4).

**Table 4:** Clustering of *agr* locus mutations among clones and *agr* genotypes

			*agrB*		*agrD*		*agrC*		*agrA*		
Strain	CC ^a^	*agr* group	SNP ^b^ (N°)	Non-synonymous predicted result	SNP (N°)	Non-synonymous predicted result	SNP (N°)	Non-synonymous predicted result	SNP (N°)	Non-synonymous predicted result	Reference
SA_123	CC45	1	35	t329c (110 Ile > Thr); g340t (114 Val > Phe); t346g (116 Ser > Ala); t349a (117 Leu > Ile); gta355ttg (119 Val > Leu); tca361att (121 Ser > Ile); gt364aa (122 Val > Lys); c452t (151 Thr > Ile); c454t (152 Leu > Phe)	5	–	26	t125g (42 Val > Gly); a752t (251 Tyr > Phe); g1249a (417 Glu > Lys)	19	–	NCTC8325
SA_520	CC45	1	30	t329c (110 Ile > Thr); g340t (114 Val > Phe); t346g (116 Ser > Ala); t349a (117 Leu > Ile); gta355ttg (119 Val > Leu); tca361att (121 Ser > Ile); gt364aa (122 Val > Lys); c452t (151 Thr > Ile); c454t (152 Leu > Phe)	5	–	25	t125g (42 Val > Gly); a752t (251 Tyr > Phe);	19	–	NCTC8325
SA_170015	CC45	1	30	t329c (110 Ile > Thr); g340t (114 Val > Phe); t346g (116 Ser > Ala); t349a (117 Leu > Ile); gta355ttg (119 Val > Leu); tca361att (121 Ser > Ile); gt364aa (122 Val > Lys); c452t (151 Thr > Ile); c454t (152 Leu > Phe)	5	–	26	t125g (42 Val > Gly); a752t (251 Tyr > Phe); t307c (103; I > T)	19	–	NCTC8325
SA_180015	CC8 ST8	1	–	–	–	–		a409 (138Lys/del -1 bp)	2	–	NCTC8325
SA_190006	CC8 ST72	1	–	–	–	–	24	t125g (42 Val > Gly); a185c (262 Asp>Ala); c773t (258 Phe > Ile)	2	–	NCTC8325
SA_70002	CC8 ST72	1	–	–	–	–	24	t125g (42 Val > Gly); a185c (262 Asp>Ala); c773t (258 Phe > Ile)	2	–	NCTC8325
SA_103	CC5	2	–	–	–	–	1	c287t (96; Ser > Leu)	–	–	N315
SA_80004	CC5	2	–	–	–	–	1	t313 (138 Phe/del -1 bp)	–	–	N315
SA_170006	CC5 ST_125	2	–	–	–	–	1	t361c (121; I > T)	–	–	N315
SA_180009	CC5 ST_125	2	–	–	–	–	–	–	–	–	N315
SA_10009	CC15	2	1	–	–	–	11	–	5	a407g (136 Lys > Arg)	N315
SA_10014	CC15	2	1	–	–	–	11	–	5	a407g (136 Lys > Arg)	N315
SA_80001	CC30	3	13	a145c (49 Ile/Leu); a343t (115 Ile/Leu)	3	–	7	g163a (55 Gly > Arg)	12	–	MW2
SA_107	CC30	3	13	a145c (49 Ile/Leu); a343t (115 Ile/Leu)	3	–	7	g163a (55 Gly > Arg)	12	–	MW2

^a^ Clonal Complex

^**b**^ Total number of single nucleotide polymorphism including synonymous and non-synonymous mutations

### Relationship between mutation profile and expression of *agr* system

**Fig. 2:**
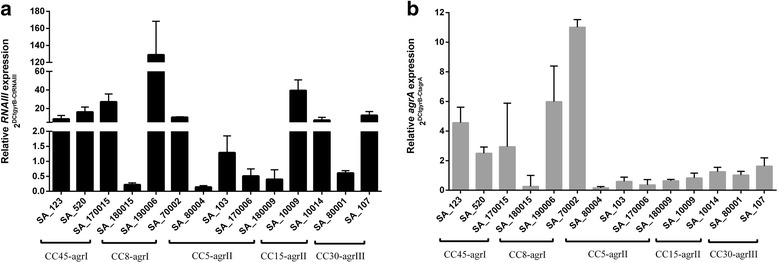
Relative expression of *RNAIII* (**a**) and *agrA* (**b**) in stationary growth phase. The expression levels of both *agrA* and *RNAIII* genes were determined by qRT–PCR experiments. The values are the means ± standard deviation (three different experiments)

After analyzing both *agrA* and *agrC* genetic profile, we aimed to investigate the potential effect of these mutations on the expression of *agr* in stationary phase. Overall there were strain-to-strain differences in the relative levels of expression (Fig. 2), which were largely independent of CC, with only strains in CC5 all exhibiting similar levels of expression. Notably, the strains in CC5 also had the overall lowest levels of *RNAIII* and *agrA* expression (*p* = 0.024 and *p* = 0.016, respectively).

Although it is difficult to determine the influence of mutations on the *agr* locus expression because of the variability found among strains, we studied whether the presence of the different mutations could have some impact on *agr* expression. In general, although some mutations detected did not seem to directly affect the expression of the *agr* system, the majority of them were associated with changes on the expression levels of *RNAIII* and/or *agrA* (Table 5). Thus, the mutation 42 Val > Gly found in the *agrC* in strains of CC45 and SA_190006 and SA_70002 of CC8 was associated with high expression levels for both *RNAIII* and *agrA* (specifically 5- and 6.9 fold, respectively)*.* Likewise, mutations 262 Asp>Ala and 258 Phe > Ile detected in SA_190006 and SA_70002 (CC8) showed a 7.3-fold increase in *RNAIII* expression levels and a 5.8-fold increase in *agrA* expression levels (*p* < 0.05). In contrast, SA_180015 (CC8, ST8) and SA_80004 (CC5) both exhibited a deletion in *agrC* exhibited 93.5- and 148-fold decrease in *RNAIII* expression levels, and 9.2- and 15.1-fold decrease in *agrA* expression values for both strains, respectively (*p* < 0.001). The other CC5 strains also had low expression values for both *RNAIII* and *agrA* and, although we did not find any deletions, there were non-synonymous mutations in *agrC* in SA_103 (96Ser > Leu) and SA_170006 (121I > T) that were associated with 15.9- and 40.3-fold decrease in *RNAIII* expression and 4.3 and 6.7 in *agrA* expression, respectively (p < 0.001). However, SA_180009 of CC5 that also exhibited low expression did not have any mutations throughout the entire *agr* operon. We observed small and nonsignificant changes in the expression of *RNAIII* by the presence of mutation detected in *agrA* (136 Lys > Arg) in strains of CC15 (*p* = 0.478). Nevertheless, this mutation was linked to low expression values for *agrA* gene with a 2.3-fold decrease (p < 0.001). Finally, for CC30 strains, the mutation detected in *agrC* (55Gly > Arg) seems to be responsible of the low means values of expression obtained for both *RNAIII* and *agrA* (3.2- and 1.80-fold decrease, respectively) (*p* < 0.05).

**Table 5:** Relationship between mutations detected and relative expression levels of *agr* locus

			*RNAIII*		*agrA*	
Mutations	Strains	CC ^a^	Absence vs Presence ^b^ Means±SD	*P* ^c^	Absence vs Presence Means±SD	P
42Val > Gly	SA_123; SA_520; SA_170015; SA_190006; SA_70002	45; 8	8.11 ± 14.27 vs. 40.22 ± 51.37	**0.001**	0.76 ± 0.53 vs. 5.26 ± 3.46	**0.000**
251Tyr > Phe	SA_123; SA_520; SA_170015	45	19.58 ± 39.28 vs. 16.90 ± 9.73	0.597	1.92 ± 3.16 vs. 3.51 ± 1.22	**0.035**
417Glu > Lys	SA_123	45	19.88 ± 36.58 vs. 8.66 ± 3.83	0.422	2.03 ± 2.96 vs. 4.57 ± 1.04	**0.019**
103I > T	SA_170015	45	18.49 ± 36.39 vs. 27.16 ± 8.56	0.563	2.21 ± 3.03 vs. 2.94 ± 0.74	0.556
138Lys/del-1 bp)	SA_180015	8	20.56 ± 36.30 vs. 0.22 ± 0.05	**0.000**	2.40 ± 2.99 vs. 0.26 ± 0.89	**0.000**
262Asp > Ala	SA_190006; SA_70002	8	10.17 ± 13.81 vs. 74.29 ± 67.51	**0.005**	1.42 ± 1.40 vs. 8.27 ± 4.01	**0.000**
258Phe > Ile	SA_190006; SA_70002	8	10.17 ± 13.81 vs. 74.29 ± 67.51	**0.005**	1.42 ± 1.40 vs. 8.27 ± 4.01	**0.000**
138Phe/del-1 bp)	SA_80004	5	20.80 ± 36.44 vs. 0.14 ± 0.04	**0.000**	2.41 ± 2.41 vs. 0.16 ± 0.85	**0.000**
96Ser > Leu	SA_103	5	20.47 ± 36.35 vs.1.29 ± 0.56	**0.000**	2.42 ± 3.02 vs 0.56 ± 0.27	**0.000**
121I > T	SA_170006	5	20.53 ± 36.31 vs. 0.51 ± 0.24	**0.000**	2.42 ± 3.00 vs 0.36 ± 0.75	**0.000**
136Lys > Arg	SA_10009; SA_10014	15	17.91 ± 37.60 vs. 25.01 ± 19.04	0.478	2.49 ± 3.16 vs 1.08 ± 0.32	**0.000**
55Gly > Arg	SA_80001; SA_107	30	20.86 ± 37.41 vs. 6.59 ± 6.82	**0.002**	2.40 ± 3.12 vs 1.33 ± 0.52	**0.007**

^a^ Clonal Complex

^b^ Values of expression are showed as relative means ± standard deviation (SD) according to the presence and absence of mutation detected relative to the *agr* group reference strains

^c^ Bold numbers indicate statistical significant results (*P* < 0.05)

## Discussion

In the current study, we found that sub-lethal concentrations of oxacillin have an impact on the levels of *agr* gene expression in a selection of clinical MSSA and MRSA strains belonging to the main clonal complexes that were associated with invasive disease in Spanish hospitals, namely CC5, CC30, CC15, CC45 and CC8 [2–4]. These differences between the presence and absence of oxacillin subMICs resulted in increases or decreases in the levels of gene expression that were strain-dependent. In addition, we observed that the strains generally shared different mutations in *agr* locus according to the CC and that these mutations had an impact on the expression values of *agr*.

A recent review has shown that sub-lethal concentrations of different antibiotics such as beta-lactams are able to modulate the expression of virulence genes [23]. Our study shows a strain-dependent effect of oxacillin subMICs on the *agr* operon expression measured as transcriptional changes in the *RNAIII* and /or *agrA* mRNA in the clinical strains included in this study. We observed an increase relative to the control condition without oxacillin in 11/14 *S. aureus* clinical strains being statically significant for 5 of them. Additionally, strains with low expression levels under exposure to oxacillin subMICs were also detected. Because of the *agr* operon plays an important role in *S. aureus* virulence the exposure to subMICs of oxacillin could have significant consequences on the pathogenesis of infection. The initiation of transcription of *RNAIII* strictly relies upon the expression of *agrA*, in a *RNAII*-dependent (*agrBDCA*) manner [8, 9, 11]. Our data support these observations since the transcription of *agrA* was observed in all strains with *RNAIII* expression. However, in the presence of oxacillin subMICs, most strains showed the same direction of change in expression values for both genes, except for 4 strains that showed divergent expression values in *RNAIII* and *agrA*, although not statistically significant. This unexpected result could be due to other factors affecting on *agr*A expression. It is important to take into account the wide network of global (*sarA, sarU, sarX, codY*) and other virulence regulators (*mgrA, sigB, saeSR*) that have a direct or indirect effect on the *agr* [35, 36], and specifically on the P2 and P3 promoters to which *agrA* binds [37], or on *agrC-agrA* interaction [38]. When we stratified the expression results by CC, we observed higher expression values of *agr* locus in presence of oxacillin subMICs than in absence of this antimicrobial agent for both CC15 and CC45. However, the relatively small number of strains affords a substantial chance for statistical bias and does not allow performing proper multivariate analyses. On the other hand, methicillin resistance has been associated with a decrease in *RNAIII* expression under exposure to suboptimal concentrations of different antibiotics [28, 34, 39]. Our results do not seem to support this hypothesis, and only one MRSA strain (SA_170006) showed a reduction in the expression values relative to the control. However, taking into account that from the 4 MRSA (*mecA* positive) strains, only one is defined as an MRSA according to CLSI breakpoints (SA_170006) [40]. This subtle difference could be impacting on the correlation MSSA/MRSA with *RNAIII/agrA* expression. Indeed, previous studies [28, 34] suggested that the expression of *mecA* and either its potential to subtly affect peptidoglycan structure or its interaction with other cell wall–associated proteins prevent the auto-inducing peptide (AIP) from being detected. These results in an unresponsive *agr* system that could be directly attributed to induced levels of *mecA* expression because of RNAIII expression was repressed as *mecA* expression increased.

Although the role of *agr* system for pathogenesis in animal model is well established [41, 42] and *agr*-defective mutants have shown an attenuated virulence and *agr* blocking agents exhibit anti-infective properties in experimental *S. aureus* infections [43], several studies have reported that *agr* defective mutants are frequently recovered from patients with bacteremia, where the mutants are associated with persistent infection and poor outcome [9, 19–21]. According to previous reports we observed that 13/14 strains harbored both synonymous and non-synonymous mutations throughout the *agr* operon, especially in *agrC* and *agrA* [9, 17, 18]*.* Moreover, we have observed that while the genotypic analysis revealed similar mutations patterns accordingly to CC, the *agr* expression was clearly strain-dependent. Some of these mutations were associated to changes in *agr* gene expression in strains with and without a specific mutation. Deletions found in SA_180015 (CC8) and SA_80004 (CC5) in *agrC* were related with low expression levels and might be responsible for reduced gene expression in these strains. Both these deletions have previously been detected in strains with dysfunctional *agr* [9, 17, 18]. Strains of CC5 exhibited the most consistent *agr* gene expression although this group had the most divergent *agr* sequences within any CC, all mutations detected in this CC were associated with decreases expression values. Finally, our observations showed that CC30 exhibited low levels of *agr* gene expression with the presence of a previously identified mutation in *agrC* [18, 44]. We recognize that the attempt to correlate *RNAIII / agrA* expression with mutations within the *agr* operon without complementation experiments is problematic, since in most cases the sample size for any given mutation was one, making it difficult to draw strong correlations between mutations and *agr* expression. Even so, these results, collectively, indicate that mutations in *agr* have an impact on *agr* expression and could be correlated with levels of *agr* gene expression for strains belonging to the same CC. Nevertheless, although different studies have shown that mutations in *agr-*defective strains do not persist in natural populations and that inactivating mutations have a relatively short life, other limitation of this study is that we do not have the results from sequencing of the *agr* operon after exposure to antibiotic, and therefore, we do not know the influence that this selective pressure could exert on mutations in the *agr* locus. Furthermore, it is possible that mutations detected in the *agr* system may have an effect not only on the expression on the *agr* locus itself, but also on other virulence genes regulated by *agr*. However, although these results could be confirmed by complementation experiments with the wild type gene, we cannot ignore the potential effect that other regulators may also have on *agr* operon expression independently of the mutations detected [24, 29]. Our findings offer a valuable starting point for further studies that analyze gene expression changes induced by oxacillin subMICs from a whole transcriptomic approach would help to provide a more accurate overview of genes and regulators that compose the virulome framework in *S. aureus* and that may be affected under this condition.

## Conclusions

We conclude that exposure to oxacillin subMICs have a stimulating or depressing effect on expression of the *agr* system for majority of the strains of *S. aureus* in our collection. In addition, the mutations we detected in the *agr* operon follow a common pattern among strains belonging to the same CC and could be responsible of changes in the *agr* expression. Consequently, the virulence mechanisms involved in the pathogenesis of infection could be affected. However, inpatient data supporting staphylococcal virulence modulation by antibiotics are rather frail, thus relevant clinical trials are necessary to extrapolate this finding to patient outcomes.

## Additional files

Additional file 1: **Figure S1.** Growth curves for *S. aureus* strains included in the study in the presence of subMIC of oxacillin. Growth was monitored by determining optical density at 600 nm (O.D600). (JPEG 5949 kb)

Additional file 2: **Table S1.** Expression levels changes in *RNAIII* and *agrA* locus under the exposure to oxacillin subMICs in the stationary growth phase. ^a^OXA: Oxacillin. ^b^POS: fold change positive in the expression level under exposure to oxacillin subMIC. NEG: fold change negative in the expression level under exposure to oxacillin subMICs. ^c^Bold numbers indicate statistical significant results (*P* < 0.05). (DOCX 19 kb)

## Abbreviations

*agr*: Accessory gene regulator
CC: Clonal complexes
MIC: Minimum inhibitory concentration
MRSA: methicillin resistant *S. aureus*
MSSA: Methicillin-susceptible *S. aureus*
OD: Optical density
SD: Standard deviation
subMICs: Sub-inhibitory concentrations
TSB: Tryptic soy broth
