# Supplementary material for: Sub-inhibitory concentrations of oxacillin modify the expression of agr locus in Staphylococcus aureus clinical strains belonging to different clonal complexes
Source: BMC Infect Dis. 2018 Apr 16;18:177. doi: 10.1186/s12879-018-3088-7 (PMC5902860; doi:10.1186/s12879-018-3088-7)
Supplement: Supplementary file 2 — Table S1. Expression levels changes in RNAIII and agrA locus under the exposure to oxacillin subMICs in the stationary growth phase. aOXA: Oxacillin. bPOS: fold change positive in the expression level under exposure to oxacillin subMIC. NEG: fold change negative in the expression level under exposure to oxacillin subMICs. cBold numbers indicate statistical significant results (P < 0.05). (DOCX 19 kb) [file 12879_2018_3088_MOESM2_ESM.docx]

**Table S1**. **Expression levels changes in *RNAIII* and *agrA* locus in absence and the presence of oxacillin subMICs in the stationary growth phase.**

|  | *RNAIII* levels expression | | | | | *agrA* levels expression | | | | |
| --- | --- | --- | --- | --- | --- | --- | --- | --- | --- | --- |
| Strain | Without OXA^a^  Means ±SD | With OXA  Means ±SD | Fold Change | | P^b,c^ | Without OXA  Means ±SD | With OXA  Means ±SD | Fold Change | | P |
| SA_123 | 8.66±3.83 | 8.04±2.33 | 1.08 | NEG | 0.735 | 4.57±1.04 | 7.82±3.05 | 1.71 | POS | 0.128 |
| SA_520 | 16.26±5.30 | 26.28±2.64 | 1.62 | POS | **0.028** | 2.50±0.42 | 4.23±0.72 | 1.69 | POS | **0.043** |
| SA_170015 | 27.16±8.56 | 57.26±21.57 | 2.11 | POS | **0.046** | 2.94±2.94 | 4.44±1.76 | 1.51 | POS | 0.116 |
| SA_180015 | 0.22±0.06 | 0.84±0.27 | 3.88 | POS | **0.028** | 0.26±0.74 | 0.43±0.14 | 1.65 | POS | 0.138 |
| SA_190006 | 128.97±39.40 | 153.73±68.09 | 1.19 | POS | 0.465 | 5.98±2.41 | 8.71±3.94 | 1.46 | POS | 0.080 |
| SA_70002 | 10.50±0.52 | 11.72±2.74 | 1.12 | POS | 0.144 | 11.01±0.52 | 21.91±6.25 | 1.99 | POS | 0.144 |
| SA_80004 | 0.14±0.05 | 0.39±0.10 | 2.90 | POS | **0.018** | 0.16±0.09 | 0.40±0.22 | 2.55 | POS | **0.046** |
| SA_103 | 1.29±0.56 | 1.71±2.53 | 1.32 | POS | 0.465 | 0.60±0.29 | 0.53±0.32 | 1.13 | NEG | 0.465 |
| SA_170006 | 0.51±0.24 | 0.14±0.01 | 3.69 | NEG | **0.043** | 0.36±0.08 | 0.40±0.08 | 1.12 | POS | 0.398 |
| SA_180009 | 0.40±0.32 | 8.23±0.36 | 20.58 | POS | 0.068 | 0.64±0.09 | 3.63±1.46 | 5.63 | POS | **0.028** |
| SA_10009 | 39.38±11.47 | 166.37±21.81 | 4.23 | POS | **0.012** | 0.83±0.33 | 7.40±2.10 | 8.91 | POS | **0.028** |
| SA_10014 | 7.47±2.98 | 10.68±3.83 | 1.43 | POS | 0.091 | 1.24±0.30 | 2.06±0.54 | 1.65 | POS | **0.018** |
| SA_80001 | 0.61±0.08 | 0.80±0.20 | 1.31 | POS | 0.249 | 1.03±0.25 | 0.77±0.31 | 1.34 | NEG | **0.028** |
| SA_107 | 12.57±4.06 | 1.04±0.29 | 12.08 | NEG | **0.028** | 1.63±0.56 | 1.19±0.62 | 1.37 | NEG | **0.345** |

^a^OXA: Oxacillin

^b^POS: fold change positive in the expression level under exposure to oxacillin subMIC. NEG: fold change negative in the expression level under exposure to oxacillin subMICs

^c^Bold numbers indicate statistical significant results (P<0.05)
